# An enhanced fractal self-pumping dressing with continuous drainage for accelerated burn wound healing

**DOI:** 10.3389/fbioe.2023.1188782

**Published:** 2023-04-04

**Authors:** Jinze Lan, Lianxin Shi, Wuyi Xiao, Xiaobin Zhang, Yuzhe Wang, Shutao Wang

**Affiliations:** ^1^ CAS Key Laboratory of Bio-inspired Materials and Interfacial Science, Technical Institute of Physics and Chemistry, University of Chinese Academy of Sciences, Beijing, China; ^2^ University of Chinese Academy of Sciences, Beijing, China; ^3^ Binzhou Institute of Technology, Weiqiao-UCAS Science and Technology Park, Binzhou City, China; ^4^ Qingdao Casfuture Research Institute Co. Ltd., Qingdao, China

**Keywords:** hollow glass microsphere, ultra-fast water absorption, self-pumping dressing, burn wound, fractal hydrogel

## Abstract

Massive exudates oversecreted from burn wounds always delay the healing process, accompanied by undesired adhesion, continuous inflammation, and high infection risk. Conventional dressings with limited draining ability cannot effectively remove the excessive exudates but constrain them in the wetted dressings immersing the wound bed. Herein, we fabricate an enhanced fractal self-pumping dressing by floating and accumulating hollow glass microspheres in the hydrogel precursor, that can continuously drain water at a non-declining high speed and effectively promote burn wound healing. Small hollow glass microspheres can split the fractal microchannels into smaller ones with higher fractal dimensions, resulting in higher absorption efficiency. In an *in vivo* burn wound model on the dorsum of murine, the enhanced fractal self-pumping dressing can significantly reduce the appearance of the wound area and alleviate tissue edema along the healing process. This study sheds light on designing high-efficiency and continuous-draining dressings for clinical applications.

## 1 Introduction

Burn injury is a typical trauma that is threatening people’s health due to its high incidence of complications, multiple organ failure (Jeschke et al., 2011; Jeschke et al., 2020; Peck and Toppi, 2020) and mortality (Chauhan and Mir, 2018). Excessive exudates secreted from the burn wounds can probably cause tissue maceration (Cutting and White, 2002), which increases the risk of infection (Glik et al., 2018) and leads to prolonged wound healing (Cook et al., 2022). Lots of absorbent dressings have been developed for absorbing wound exudates to promote healing, such as polyacrylate fibers (Tyan et al., 2003; Desroche et al., 2016), chitosan foam (Loke et al., 2000), and bio-ceramic powder (Zhang Z. W. B. et al., 2021). However, the current dressings cannot effectively remove residual exudates remained at the tissue-dressing interface, usually resulting in secondary injuries (Li et al., 2019; Shi et al., 2019), prolonged healing process and infected wounds (Church et al., 2006).

Hydrogel dressings, due to their unique porous structure and swelling properties, can effectively absorb wound exudates and provide a moist environment for wound healing (Lee and Mooney, 2012; Calo and Khutoryanskiy, 2015; Guo et al., 2021; Kharaziha et al., 2021; Montazerian et al., 2022). Various approaches have been developed for improving the water absorption capacity of hydrogels, such as grafting high water-absorbing polymer molecules and constructing oriented internal structures (Leonhardt et al., 2019; Wang et al., 2019; Li et al., 2020). Further, self-pumping dressings with asymmetric wettability were developed to directionally drain exudates. The air-side hydrophilic microfibers offer draining force, while the skin-side hydrophobic nanofiber array ensures its directional penetration (Shi et al., 2019). Based on this concept, some ingenuous self-pumping dressings have been developed with diverse functions for promoting wound healing, such as antibacterial and antioxidant properties (Zhang K. et al., 2021; Qi et al., 2021), bioactive ion releasing (Bao et al., 2020), and anti-adhesion (Luo et al., 2021). However, the above hydrogels or self-pumping dressings may not be adequate to remove the continuous exudates from burn wounds. It is an urgent need to develop novel dressings with high drainage efficiency that can promote healing and reduce the physical and psychological suffering of patients with burn wounds.

The design of fractal structure whose local structure is highly similar to the overall structure (Martin and Hurd, 1987) can increase the liquid transportation efficiency of draining model (Mauroy et al., 2004; Zhou et al., 2012; Poyet, 2021). In a given space, fractal structures can provide higher capillary pressure, shorter time of capillary flow (Shou et al., 2014) and faster liquid flow rate (Lee et al., 2020) in absorbing water compared to equivalent parallel capillary tube (Fan et al., 2015). Thus, further exploration is needed on how to design fractal structures in wound dressings that can meet clinical application requirements of removing continuous exudates from burn wounds.

In this study, we prepare an enhanced fractal self-pumping hydrogel (EFS hydrogel) dressing with ultra-fast water absorption and continuous drainage by a simple floating-accumulating process. Hollow glass microspheres (HGM) are introduced into the hydrogel precursor to form a fractal arrangement that diameter decreases along the gravity direction. Then a series of connected fractal hydrophilic microchannels with gradually decreased diameters are fabricated between HGM after crosslinking the hydrogel. The synthesized EFS hydrogel with fractal hydrophilic microchannels exhibits ultra-fast and continuous water absorbing performance. In the burn wound model on murine, the EFS hydrogel exhibits accelerated healing compared to the pure hydrogel, commercial Tegaderm™ dressing and gauze. The unique design of the enhanced fractal self-pumping dressing has great potential in developing biofluid management materials especially for those highly exudated wounds.

## 2 Materials and methods

### 2.1 Reagents and materials

Acrylamide (AAm), N,N-Methylene bisacrylamide (MBAA), 2,2-Diethoxyacetophenone (DEAP), Sodium alginate (SA) was purchased from Macklin reagent (China). All the regents were used directly without further purification. Rhodamine 123 (Rh123, J&K Scientific, China) were used for fluorescence staining. HGM was supported by Research and Development Center of Oil and Gas Development and New Materials for energy conservation and environmental protection, Institute of Physics and Chemistry, China. Commercial dressing Tegaderm™ film was obtained from 3M Healthcare (United States).

### 2.2 Preparation of the EFS hydrogel

The EFS hydrogel was prepared by adjusting the floating-accumulating process of HGM in hydrogel precursor solution. Hydrogel precursor solution: AAm (10 g) was dissolved in deionized water (100 mL), and then MBAA (0.1 g), and DEAP (0.1 g) were added to the hydrogel precursor solution respectively. To increase the viscosity, 2 g of SA was added to the hydrogel precursor solution. The hydrogel precursor and HGM were mixed at a volume ratio of 8:2 and dispersed by ultrasound at 150 W power through an ultrasonic cell breaker (JY92-IIN, Ningbo Kezhi Biotechnology Co., LTD., China). The precursor solution was allowed to stand for 30 min at room temperature to allow the HGM to float up and form a microspheres layer before the cross-linking step. They were then photocrosslinked under 365 nm UV for 20 min and freeze-dried to obtain the final sample. To investigate the effect of the enhanced fractal structure on the absorption performance, HGM with different diameter distributions, diameter 5–80 μm as enhanced fractal microchannels and 20–80 μm as normal fractal microchannels, were used for comparison.

### 2.3 Evaluation of continuous water absorption capability

The dynamic contact angles (CA) of water droplets on the samples (1 × 1 cm^2^) were recorded by the OCA-20 machine (Dataphysics Germany). The volume of each water droplets was set as 2 μL.

### 2.4 Calculation of fractal dimension

The fractal dimension results are calculated by ‘Fractal box count’, a plug-in of software ‘ImageJ 1.50i’ (National Institutes of Health, United States).

### 2.5 *In vivo* burn wound model

8-week-old female Sprague Dawley (SD) rats were used for burn wound models (n = 5). After depilating the back of rats, four burn wounds were produced on the back of each rat under anesthesia. The desktop super temperature control scald device (YLS-5Q, Beijing, China) was employed to control the wound area (0.8 cm^2^), burn time (5 s), temperature (100°C) and pressure (200 g). Then EFS hydrogel, pure hydrogel, Tegaderm™ (3M, United States), and gauze were immobilized on the wound sites with 3M Micropore™ tape and replaced every 2 days.

## 3 Results and discussion

**FIGURE 1:**
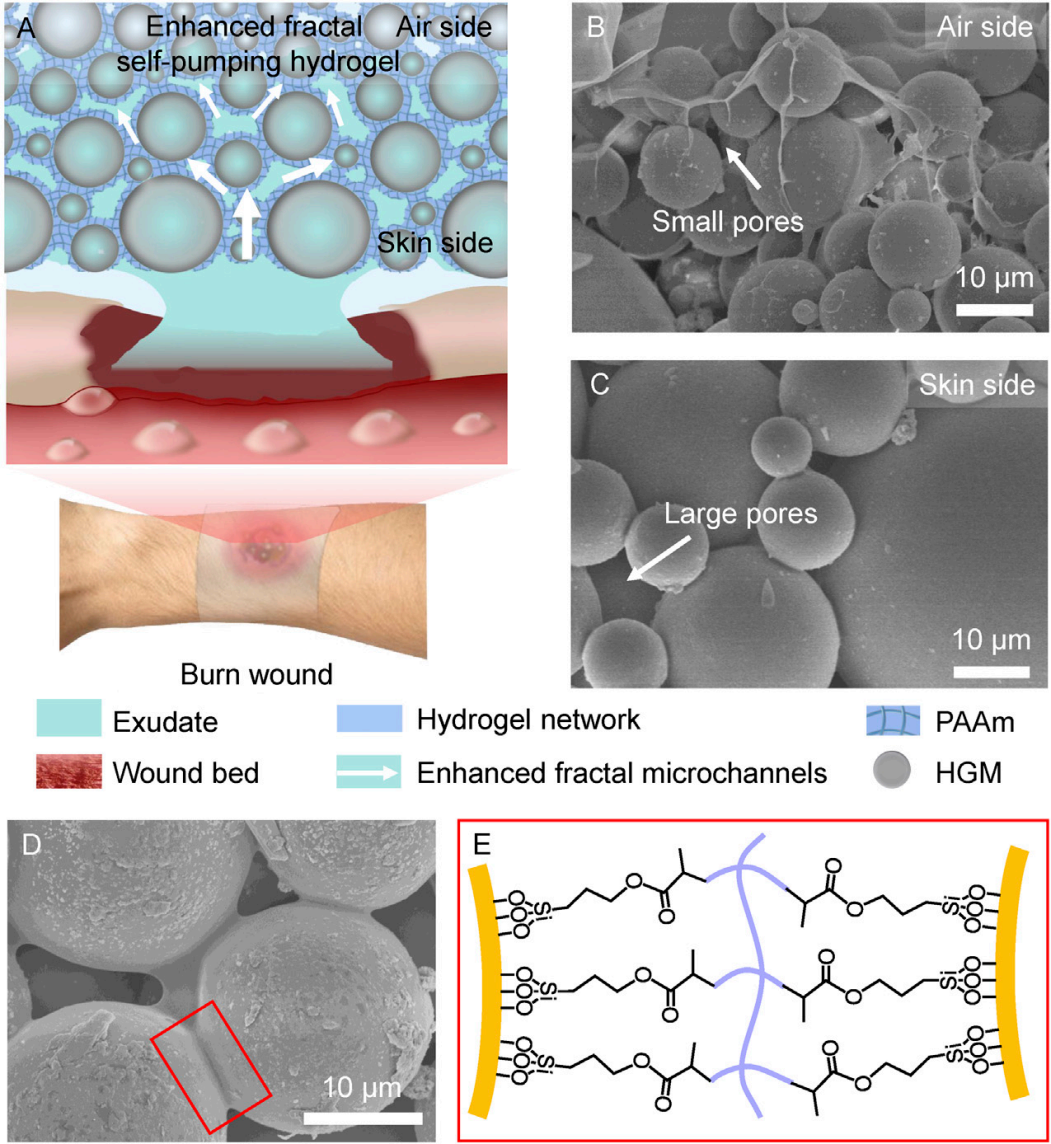
Design of the enhanced fractal self-pumping (EFS) hydrogel dressing. **(A)** Illustration of the EFS hydrogel with enhanced fractal microchannels for draining exudates from burn wounds. **(B, C)** SEM images of the gradient HGM with small pores at air side **(B)** and large pores at skin side **(C)** in the EFS hydrogel. **(D)** SEM image of the hydrogel network between the adjacent HGM. **(E)** Schematic graph of connection between HGM and hydrogel network by silane coupling agent.

The design of enhanced fractal self-pumping hydrogel (EFS hydrogel) dressing is illustrated in Figure 1A that can remove excessive exudates from burn wounds through the enhanced fractal microchannels. In the EFS hydrogel, hollow glass microspheres (HGM) with diameter of 5–80 μm were embedded in polyacrylamide (PAAm) hydrogel network (blue frame) with a gradually decreased diameter from skin side to air side (as shown in Figures 1B, C). Different with the normal fractal structure, small HGM are inset into gaps between the adjacent large HGM in the EFS hydrogel (Figures 1B, C). By squeezing the hydrogel network, the enhanced fractal hydrophilic microchannels of hydrogel were formed between HGM (Figure 1D). The hydrogel network was anchored onto the surface of silane coupling agent modified HGM through C=C bond cross-linking (Figure 1E). The unique enhanced fractal self-pumping microchannels were successfully fabricated in EFS hydrogel.

The fractal structure was formed by the accumulation of floating HGM. When HGM whose density is lower than that of the hydrogel precursor solution is dispersed in the hydrogel precursor solution, it would float driven by the buoyancy. During this upward movement, it follows the Stokes law. The displacement velocity $V_{Stokes}$ can be calculated using the Stokes settling formula (Goodarzi and Zendehboudi, 2019):

$$V_{Stokes}=\frac{\left(\rho_{H}-\rho_{w}\right)gd^{2}}{18\mu_{w}}$$ (1)

Here $\rho_{H}$ and $\rho_{w}$ refer the densities of HGM and water phase, respectively. d refers to the HGM diameter. g refers to the acceleration of gravity. $\mu_{w}$ refers to the viscosity of the water phase.

According to Eq. 1, under the conditions of constant HGM density and water-related factors, larger diameter of HGM would increase its floating velocity ( $V_{Stokes}$ ). High $V_{Stokes}$ will drive large HGM to accumulate near the air-liquid interface, resulting in a fractal arrangement of HGM that diameter decreases along the gravity direction.

**FIGURE 2:**
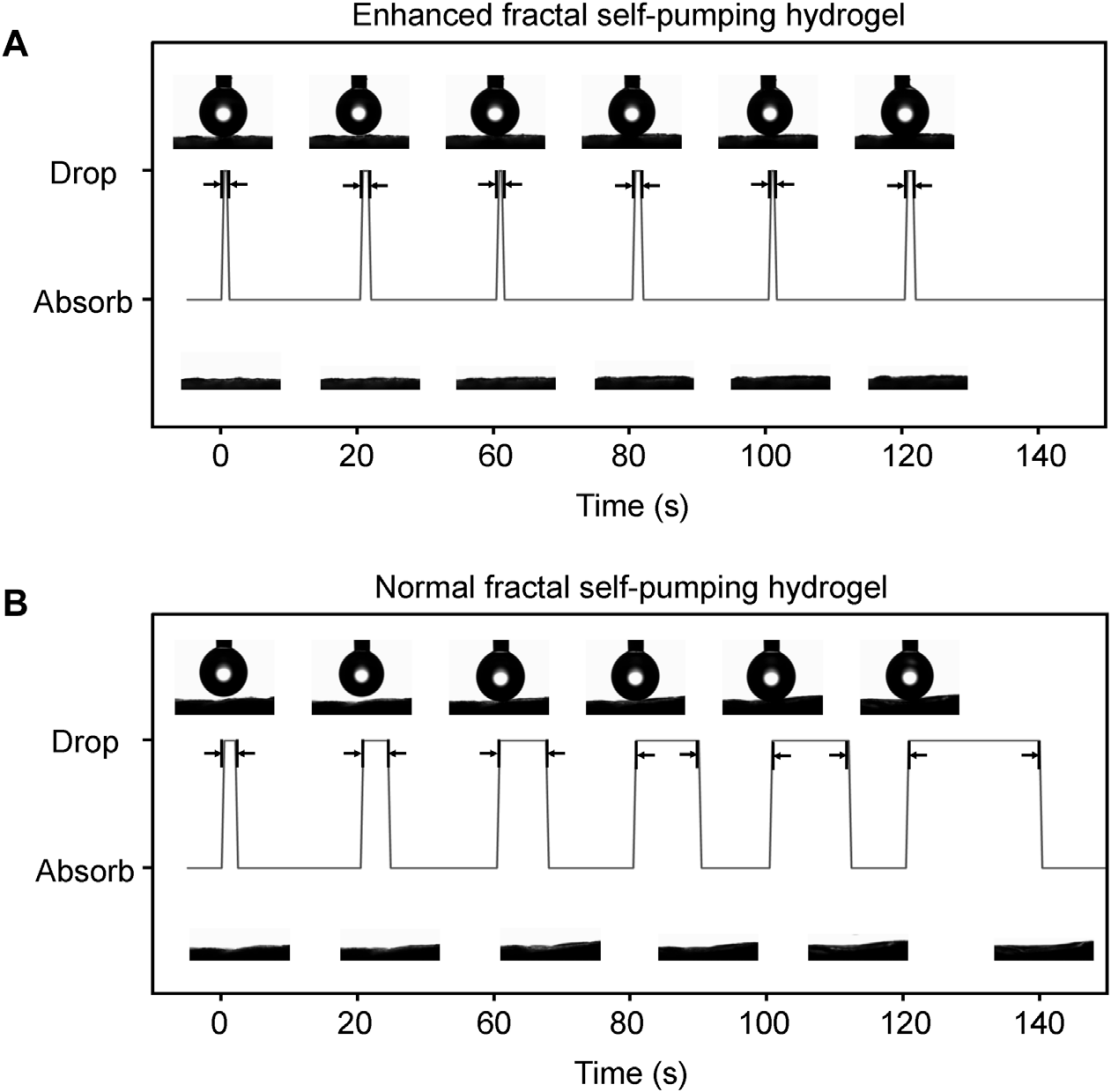
Comparison of the continuous absorption performance of the EFS hydrogel **(A)** with enhanced fractal microchannels and the normal fractal self-pumping hydrogel **(B)** without enhanced fractal microchannels.

Further, we compared the dynamic contact angles of the continuous water dripping on HGM with the diameter distribution of 5–80 μm (enhanced fractal channels) and that of 20–80 μm (normal fractal channels). EFS hydrogels (Figure 2A) had a fast water absorption (initially 0.4 s) and no attenuation of water absorption performance during continuous absorption. In contrast, normal fractal self-pumping hydrogel (Figure 2B) had a slow water absorption (initially 1.6 s) and a significant increase in absorption time with continuous drip of water droplets (increasing to 19.2 s). The videos of the continuous water absorption performance of EFS hydrogel (Supplementary Video S1) and normal fractal self-pumping hydrogel (Supplementary Video S2) were included in the Supplementary Videos (The videos were played at 20x speed).

**FIGURE 3:**
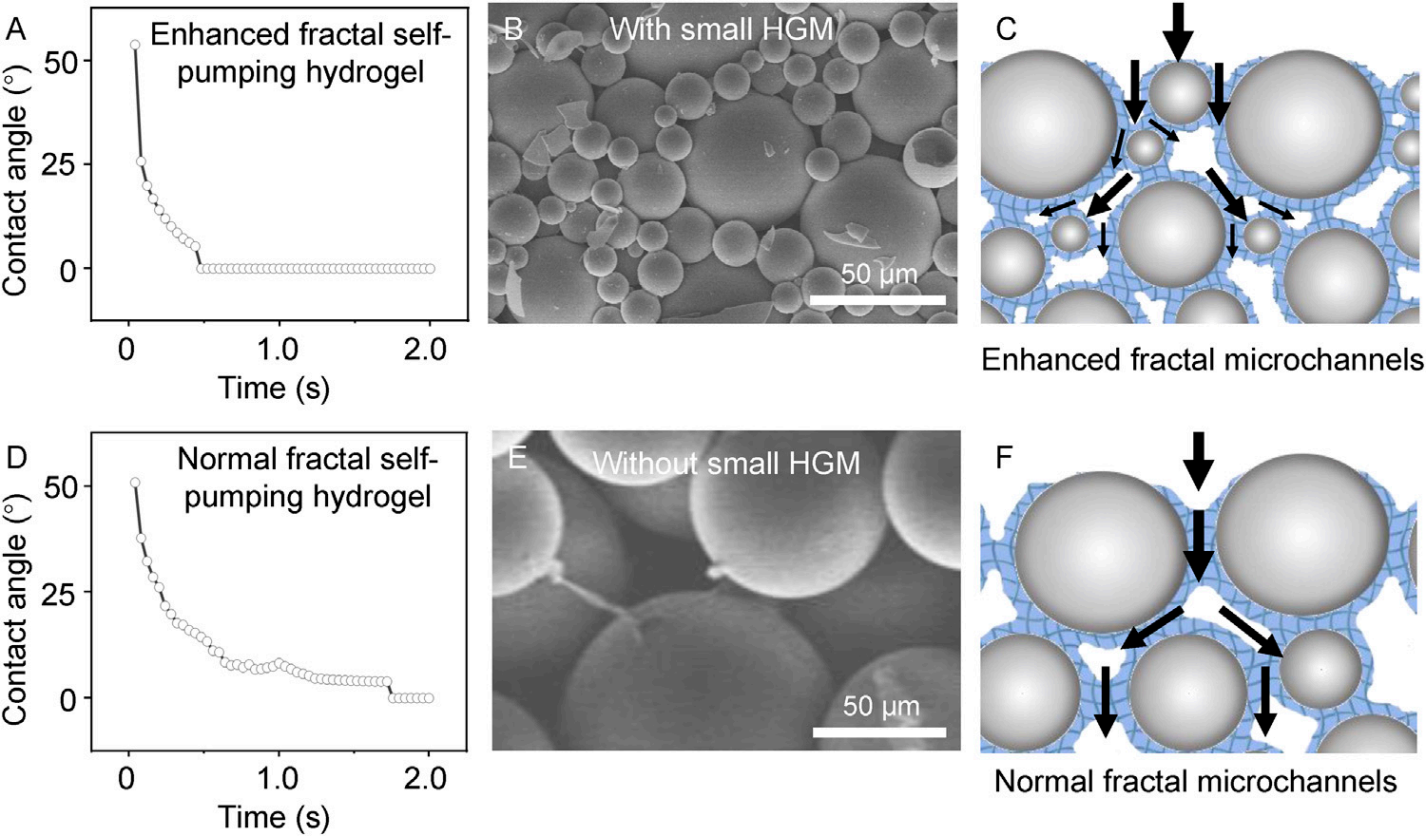
Comparison of the EFS hydrogel **(A–C)** and normal fractal self-pumping hydrogel **(D–F)** without enhanced fractal microchannels in absorption rate **(A, D)**, structure **(B, E)**, and draining model **(C, F)**.

To explore the improvement in water absorption performance, we characterized the microstructures of two types of fractal self-pumping hydrogels (Figure 3). The EFS hydrogel can absorb water droplet (2 μL) within 0.4 s (Figure 3A), while normal fractal self-pumping hydrogel takes 1.6 s (Figure 3D). The reason of the ultra-fast absorption is that the enhanced fractal microchannels in EFS hydrogel are splitted into smaller ones by the small HGM, resulting in a larger fractal dimension. The large fractal dimension value indicates a more complex microchannel structure and higher conductivity efficiency (Xu et al., 2019). This theoretical deduction is consistent with the experimental result that EFS samples with a wider diameter distribution exhibit higher water absorption efficiency.

**FIGURE 4:**
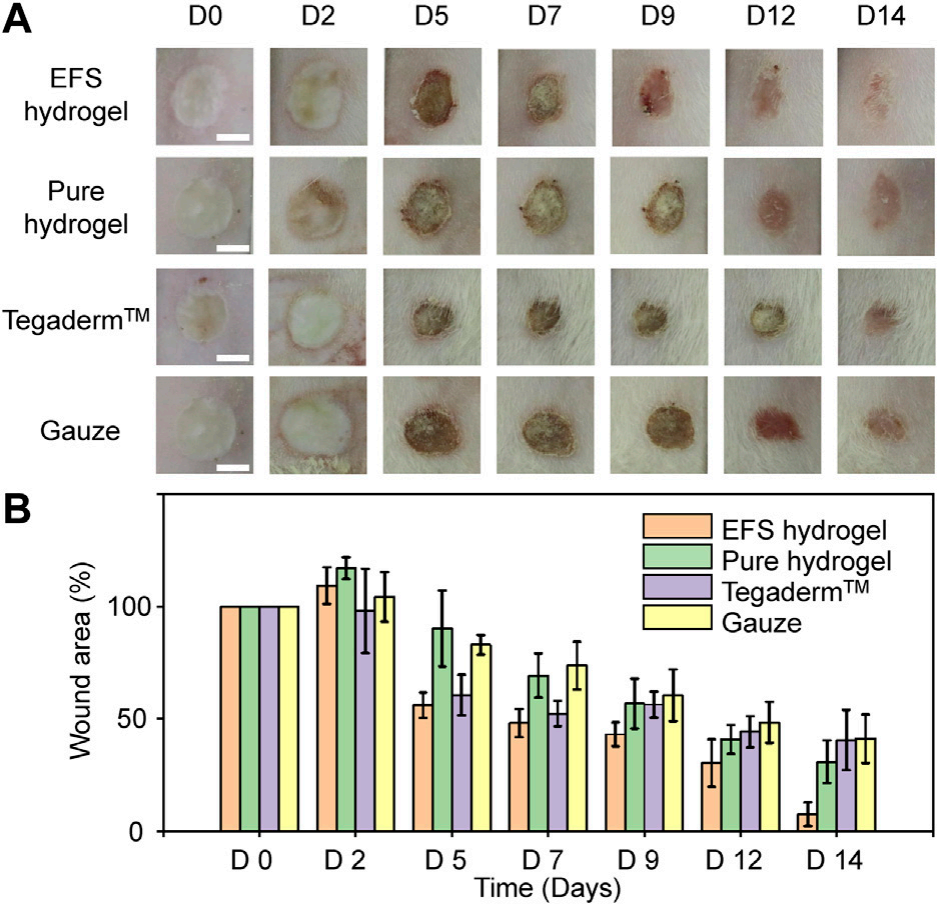
Burn healing performance treated by various dressings. **(A)** Digital photos of burn wound after 0, 2, 5, 7, 9, 12 and 14 days treatment by EFS hydrogel, pure hydrogel, commercial Tegaderm™ dressing, and gauze, respectively. **(B)** Statistical wound areas of the corresponding treatment during 14 days.

To verify the promoting effect of EFS hydrogel on wound healing, we explored the performance of EFS hydrogel dressings in the burn wound model on murine. We used the pure hydrogel, commercial dressing (Tegaderm™), and gauze as controls. According to the digital photos of the wound appearance in Figure 4, the edema of wounds treated with EFS hydrogel were alleviated compared to the other three groups on the second day, reflecting better draining of exudates. On the 5th day, the wound area of the EFS hydrogel group was significantly reduced. On the 9th day, the scab on the wound surface almost disappeared. It completed the decrustation process on the 14th day treated by the EFS hydrogel. Wound area quantification was conducted at days 2, 5, 7, 9, 12, and 14 after injury (Figure 4B). The reduction rate wound areas of EFS hydrogel treatment are significantly higher than those of other groups. Based on these results, EFS hydrogel can effectively promote burn wound healing compared to the other three treatments.

## 4 Conclusion

In conclusion, we have successfully fabricated an enhanced fractal self-pumping (EFS) hydrogel dressing with enhanced fractal microchannels constructed through a simple buoyancy-driven floating-accumulating process that can absorb water in a high absorption efficiency continuously and accelerate burn wound healing. The enhanced fractal microchannels are formed between the adjacent HGM by squeezing the hydrogel networks. The as synthesized EFS hydrogel possess enhanced draining ability that can absorb water within 0.4 s and continuously drain water without any decreasing efficiency. In the murine burn wound model, the EFS hydrogel exhibits accelerated wound healing. Enhanced fractal microchannels create a new construction model for fabricating functional materials with ultra-fast draining ability. This study will open a new avenue for managing continuous biofluids in clinical research and applications.

## Data Availability

The raw data supporting the conclusion of this article will be made available by the authors, without undue reservation.

## References

[B1] Bao F. Pei G. Wu Z. Zhuang H. Zhang Z. Huan Z. (2020). Bioactive self‐pumping composite wound dressings with micropore array modified janus membrane for enhanced diabetic wound healing. Adv. Funct. Mater. 30 (49), 2005422. 10.1002/adfm.202005422

[B2] Calo E. Khutoryanskiy V. V. (2015). Biomedical applications of hydrogels: A review of patents and commercial products. Eur. Polym. J. 65, 252–267. 10.1016/j.eurpolymj.2014.11.024

[B3] Chauhan V. S. Mir M. A. (2018). Plaster burn: Challenge to plastic surgeon. World J. plastic Surg. 7 (2), 226–230. PMC6066717 30083507

[B4] Church D. Elsayed S. Reid O. Winston B. Lindsay R. (2006). Burn wound infections. Clin. Microbiol. Rev. 19 (2), 403–434. 10.1128/cmr.19.2.403-434.2006 16614255 PMC1471990

[B5] Cook K. A. Martinez-Lozano E. Sheridan R. Rodriguez E. K. Nazarian A. Grinstaff M. W. (2022). Hydrogels for the management of second-degree burns: Currently available options and future promise. Burns trauma 10, tkac047. 10.1093/burnst/tkac047 36518878 PMC9733594

[B6] Cutting K. F. White R. J. (2002). Maceration of the skin and wound bed. 1: Its nature and causes. J. wound care 11 (7), 275–278. 10.12968/jowc.2002.11.7.26414 12192848

[B7] Desroche N. Dropet C. Janod P. Guzzo J. (2016). Antibacterial properties and reduction of MRSA biofilm with a dressing combining polyabsorbent fibres and a silver matrix. J. Wound Care 25 (10), 577–584. 10.12968/jowc.2016.25.10.577 27681588

[B8] Fan J. Zhu N. Liu Z. Cheng Q. Liu Y. (2015). A model for allometric permeation in fractal branching channel net driven by capillary pressure. Int. J. Numer. Methods Heat Fluid Flow 25 (8), 1886–1895. 10.1108/hff-01-2014-0011

[B9] Glik J. Labus W. Kitala D. Mikus-Zagorska K. Roberts C. D. Nowak M. (2018). A 2000 patient retrospective assessment of a new strategy for burn wound management in view of infection prevention and treatment. Int. Wound J. 15 (3), 344–349. 10.1111/iwj.12871 29243368 PMC7949883

[B27] Goodarzi F. Zendehboudi S. (2019). A comprehensive review on emulsions and emulsion stability in chemical and energy industries. Can. J. Chem. Eng. 97 (1), 281–309. 10.1002/cjce.23336

[B10] Guo B. L. Dong R. N. Bang Y. P. Li M. (2021). Haemostatic materials for wound healing applications. Nat. Rev. Chem. 5 (11), 773–791. 10.1038/s41570-021-00323-z 37117664

[B11] Jeschke M. G. Gauglitz G. G. Kulp G. A. Finnerty C. C. Williams F. N. Kraft R. (2011). Long-term persistance of the pathophysiologic response to severe burn injury. PLoS ONE 6 (7), e21245. 10.1371/journal.pone.0021245 21789167 PMC3138751

[B12] Jeschke M. G. van Baar M. E. Choudhry M. A. Chung K. K. Gibran N. S. Logsetty S. (2020). Burn injury. Nat. Rev. Dis. Prim. 6 (1), 11. 10.1038/s41572-020-0145-5 32054846 PMC7224101

[B13] Kharaziha M. Baidya A. Annabi N. (2021). Rational design of immunomodulatory hydrogels for chronic wound healing. Adv. Mater. 33 (39), 2100176. 10.1002/adma.202100176 PMC8489436 34251690

[B14] Lee J. J. Berthier J. Kearney K. E. Berthier E. Theberge A. B. (2020). Open-Channel capillary trees and capillary pumping. Langmuir 36 (43), 12795–12803. 10.1021/acs.langmuir.0c01360 32936651 PMC8259885

[B15] Lee K. Y. Mooney D. J. (2012). Alginate: Properties and biomedical applications. Prog. Polym. Sci. 37 (1), 106–126. 10.1016/j.progpolymsci.2011.06.003 22125349 PMC3223967

[B16] Leonhardt E. E. Kang N. Hamad M. A. Wooley K. L. Elsabahy M. (2019). Absorbable hemostatic hydrogels comprising composites of sacrificial templates and honeycomb-like nanofibrous mats of chitosan. Nat. Commun. 10, 2307. 10.1038/s41467-019-10290-1 31127114 PMC6534699

[B17] Li D. W. Bu X. C. Xu Z. P. Luo Y. W. Bai H. (2020). Bioinspired multifunctional cellular plastics with a negative Poisson's ratio for high-energy dissipation. Adv. Mater. 32 (33), 2001222. 10.1002/adma.202001222 32644270

[B18] Li Z. Milionis A. Zheng Y. Yee M. Codispoti L. Tan F. (2019). Superhydrophobic hemostatic nanofiber composites for fast clotting and minimal adhesion. Nat. Commun. 10, 5562. 10.1038/s41467-019-13512-8 31804481 PMC6895059

[B19] Loke W. K. Lau S. K. Yong L. L. Khor E. Sum C. K. (2000). Wound dressing with sustained anti-microbial capability. J. Biomed. Mater. Res. 53 (1), 8–17. 10.1002/(sici)1097-4636(2000)53:1<8:aid-jbm2>3.0.co;2-3 10634947

[B20] Luo Z. Jiang L. Xu C. F. Kai D. Fan X. S. You M. L. (2021). Engineered Janus amphipathic polymeric fiber films with unidirectional drainage and anti-adhesion abilities to accelerate wound healing. Chem. Eng. J. 421, 127725. 10.1016/j.cej.2020.127725

[B21] Martin J. E. Hurd A. J. (1987). Scattering from fractals. J. Appl. Crystallogr. 20, 61–78. 10.1107/s0021889887087107

[B22] Mauroy B. Filoche M. Weibel E. R. Sapoval B. (2004). An optimal bronchial tree may be dangerous. Nature 427 (6975), 633–636. 10.1038/nature02287 14961120

[B23] Montazerian H. Davoodi E. Baidya A. Baghdasarian S. Sarikhani E. Meyer C. E. (2022). Engineered hemostatic biomaterials for sealing wounds. Chem. Rev. 122, 12864–12903. 10.1021/acs.chemrev.1c01015 35731958

[B24] Peck M. D. Toppi J. T. (2020). Handbook of burns volume 1 acute burn Care. 2nd Edition. Switzerland: Springer.

[B25] Poyet S. (2021). Water transport properties of virtual fractal porous media: Implications for the unsaturated transport properties of cement-based materials. Cem. Concr. Res. 150, 106613. 10.1016/j.cemconres.2021.106613

[B26] Qi L. Y. Ou K. K. Hou Y. J. Yuan P. P. Yu W. Li X. (2021). Unidirectional water-transport antibacterial trilayered nano fi ber-based wound dressings induced by hydrophilic-hydrophobic gradient and self-pumping effects. Mater. Des. 201, 109461. 10.1016/j.matdes.2021.109461

[B28] Shi L. X. Liu X. Wang W. S. Jiang L. Wang S. T. (2019). A self-pumping dressing for draining excessive biofluid around wounds. Adv. Mater. 31 (5), 1804187. 10.1002/adma.201804187 30537340

[B29] Shou D. H. Ye L. Fan J. T. (2014). Treelike networks accelerating capillary flow. Phys. Rev. E 89 (5), 053007. 10.1103/PhysRevE.89.053007 25353880

[B30] Tyan Y. C. Liao J. D. Lin S. P. (2003). Surface properties and *in vitro* analyses of immobilized chitosan onto polypropylene nonwoven fabric surface using antenna-coupling microwave plasma. J. Mater. Science-Materials Med. 14 (9), 775–781. 10.1023/a:1025036421604 15348397

[B31] Wang C. W. Niu H. Y. Ma X. Y. Hong H. Yuan Y. Liu C. S. (2019). Bioinspired, injectable, quaternized hydroxyethyl cellulose composite hydrogel coordinated by mesocellular silica foam for rapid, noncompressible hemostasis and wound healing. ACS Appl. Mater. Interfaces 11 (38), 34595–34608. 10.1021/acsami.9b08799 31464418

[B32] Xu J. Wu K. Li R. Li Z. Li J. Xu Q. (2019). Nanoscale pore size distribution effects on gas production from fractal shale rocks. Fractals 27 (08), 1950142. 10.1142/s0218348x19501421

[B33] Zhang K. Jiao X. Zhou L. Wang J. Wang C. Qin Y. (2021). Nanofibrous composite aerogel with multi-bioactive and fluid gating characteristics for promoting diabetic wound healing. Biomaterials 276, 121040. 10.1016/j.biomaterials.2021.121040 34352626

[B34] Zhang Z. W. B. Li W. B. Liu Y. Yang Z. G. Ma L. L. Zhuang H. (2021). Design of a biofluid-absorbing bioactive sandwich-structured Zn-Si bioceramic composite wound dressing for hair follicle regeneration and skin burn wound healing. Bioact. Mater. 6 (7), 1910–1920. 10.1016/j.bioactmat.2020.12.006 33364530 PMC7750441

[B35] Zhou G. N. Simerly T. Golovko L. Tychinin I. Trachevsky V. Gomza Y. (2012). Highly functionalized bridged silsesquioxanes. J. Sol-Gel Sci. Technol. 62 (3), 470–482. 10.1007/s10971-012-2751-5

